# Identifying tumor cell-released extracellular vesicles as biomarkers for breast cancer diagnosis by a three-dimensional hydrogel-based electrochemical immunosensor

**DOI:** 10.1186/s12951-023-02180-y

**Published:** 2023-12-07

**Authors:** Yue Zhang, Deng Pan, Zhenqiang Ning, Fang Huang, Yiting Wei, Mingming Zhang, Yuanjian Zhang, Li-xin Wang, Yanfei Shen

**Affiliations:** 1 Clinical Medical Laboratory Center, The Affiliated Taizhou People’s Hospital of Nanjing Medical University, Taizhou, 225300 China; 2 Medical School, Southeast University, Nanjing, 210009 China; 3 School of Chemistry and Chemical Engineering, Southeast University, Nanjing, 211189 China

**Keywords:** Biomarker, LC3^+^ extracellular vesicles, Breast cancer, Early diagnosis, cancer subtypes and stages

## Abstract

Tumor cell-released LC3^+^ extracellular vesicles (LC3^+^ EVs) participate in immunosuppression during autophagy and contribute to the occurrence and development of breast cancer. In view of the strong association between the LC3^+^ EVs and breast cancer, developing an effective strategy for the quantitative detection of LC3^+^ EVs levels with high sensitivity to identify LC3^+^ EVs as new biomarkers for accurate diagnosis of breast cancer is crucial, but yet not been reported. Herein, an ultrasensitive electrochemical immunosensor is presented for the quantitative determination of LC3^+^ EVs using a three-dimensional graphene oxide hydrogel-methylene blue composite as a redox probe, showing a low detection limit and a wide linear range. With this immunosensor, the expression levels of LC3^+^ EVs in various practical sample groups including different cancer cell lines, the peripheral blood of tumor-bearing mice before and after immunotherapy, and the peripheral blood from breast cancer patients with different subtypes and stages were clearly distinguished. This study demonstrated that LC3^+^ EVs were superior as biomarkers for the accurate diagnosis of breast cancer compared to traditional biomarkers, particularly for cancer subtype discrimination. This work would provide a new noninvasive detection tool for the early diagnosis and prognosis assessment of breast cancer in clinics.

**Supplementary Information:**

The online version contains supplementary material available at 10.1186/s12951-023-02180-y.

## Introduction

Breast cancer, often called “pink killer”, is the largest threat to women’s health, with high morbidity and mortality all over the world [1–4]. The latest data released by the International Agency for Research on Cancer (IARC) showed that the number of new cases of breast cancer has surpassed that of lung cancer until 2021, becoming the most common cancer worldwide [5]. The five-year survival rates of stage I and stage IV breast cancer are 99% and 29%, respectively [6, 7]. In this sense, the early diagnosis of breast cancer plays a critical role in improving the survival rate of patients. Like other cancer types, the current gold standard for breast cancer diagnosis is pathological biopsy, which requires invasive access to the tumor [8, 9]. Alternatively, liquid biopsy, a kind of non-invasive testing, provides the opportunity to monitor cancer in various body effluents such as blood or urine instead of a fragment of tumor tissues [10–13]. The carcinoembryonic antigen (CEA) and carcinoma antigen 15 − 3 (CA15-3) are usually employed as tumor markers for the diagnosis and evaluation of the progress of breast cancer [14–16]. However, benign breast disease can also associate with increased levels of CEA and CA15-3, leading to low specificity of CEA and CA15-3 [17, 18]. Therefore, identifying a novel tumor marker with high specificity is highly desirable for early diagnosis of breast cancer.

Autophagy is an essential cellular process in cancer, including breast cancer, during which LC3^+^ extracellular vesicles (LC3^+^ EVs), nano-sized vesicles, are released by tumor cells [19–21]. It has been reported that tumor cell-released LC3^+^ EVs have an impact on the functions of immune cells and play a crucial role in the occurrence and development of cancer, particularly breast cancer [22–27]. Since LC3^+^ EVs exist in body fluids and exhibit specific immunologic functions, LC3^+^ EVs have emerged as potential biomarkers for cancer diagnosis at an early stage and prognosis. LC3^+^ EVs are increasingly being favored as biomarkers over exosomes. This is because LC3^+^ EVs have a unique and well-defined surface marker, LC3, which makes them easier to identify and isolate. Additionally, LC3^+^ EVs are larger and more abundant in peripheral blood than exosomes, which makes them easier to detect. Therefore, a facile method for effective separation and quantitative analysis of LC3^+^ EVs plays a vital role in biomarker applications. Due to the extremely low abundance of LC3^+^ EVs in body fluids, differential ultracentrifugation has been usually applied for the separation and enrichment of LC3^+^ EVs, which requires special and expensive instruments with cumbersome operations [28, 29].

As for the detection of LC3^+^ EVs, several typical techniques, including transmission electron microscope (TEM), western blot, and flow cytometry, have been developed [30–32]. However, these techniques can only be used for qualitative or semi-quantitative detection of LC3^+^ EVs. Thus, there is still a lack of simple yet effective strategies for the separation and quantitative analysis of LC3^+^ EVs, limiting the utility of LC3^+^ EVs as routine biomarkers in clinics. Therefore, developing facile strategies for the effective capture and highly sensitive biosensing of LC3^+^ EVs is highly desirable.

**Fig. 1:**
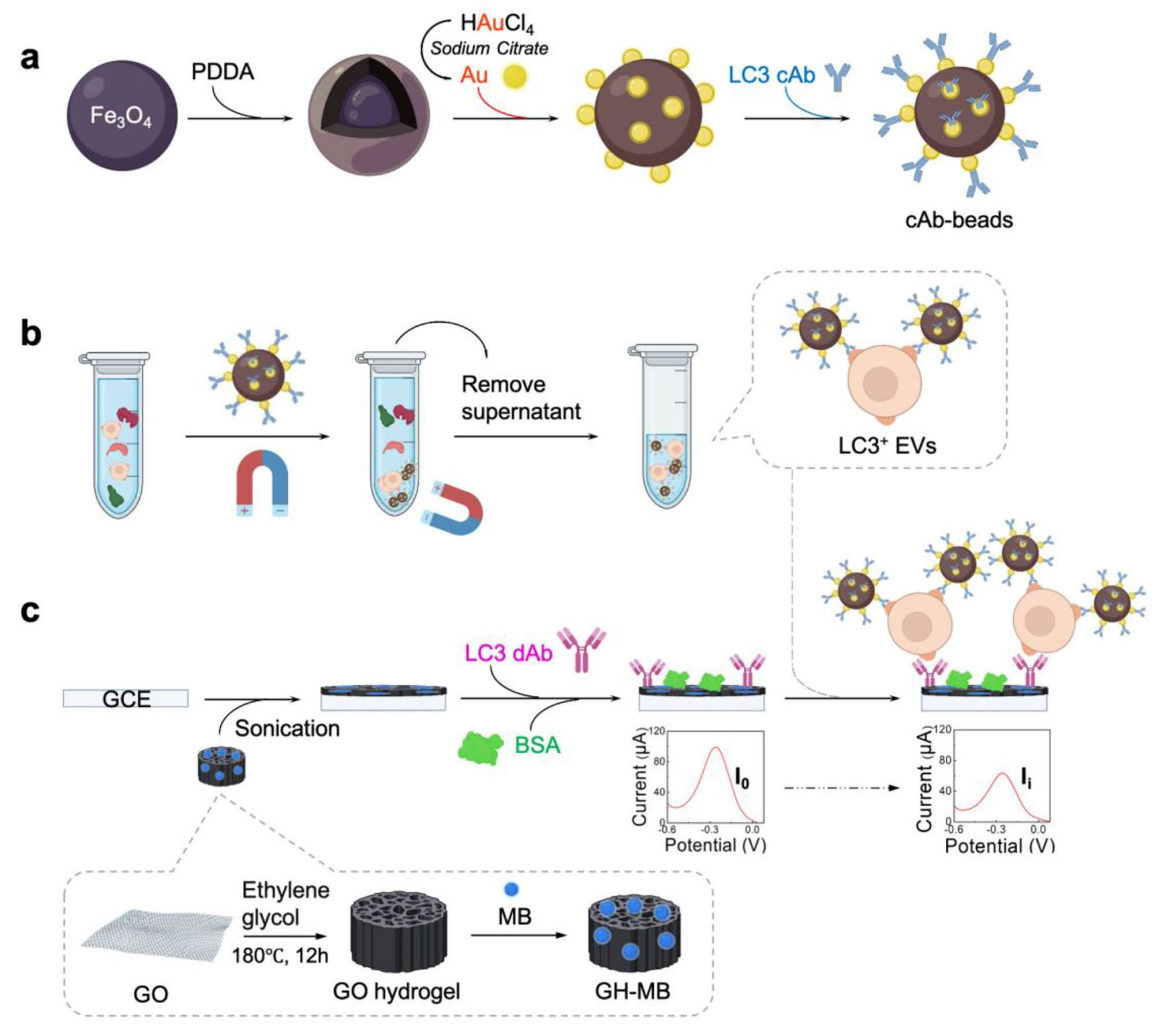
Illustration of the electrochemical immunosensor for LC3^+^ EVs. (a) Synthesis of cAb-beads. (b) Enrichment of LC3^+^ EVs with cAb-beads. (c) Fabrication process of immunosensor. (LC3 cAb: LC3 capture antibody; LC3 dAb: LC3 detection antibody). Created with BioRender.com

Herein, we report a two-step strategy to construct an electrochemical immunosensor for the determination of LC3^+^ EVs using immunomagnetic beads for LC3^+^ EVs enrichment (named cAb-beads) based on immunoaffinity capture and employing graphene oxide hydrogel-methylene blue (GH-MB) as a redox probe (Fig. 1). The proposed immunosensor displayed a wide calibration range with a low detection limit of 15.7 pg mL^− 1^. With the as-constructed electrochemical immunosensor, the LC3^+^ EVs levels in actual samples, including tumor cell supernatants, peripheral blood from tumor-bearing (TB) mouse models, and breast cancer patients were determined. The results showed that the constructed immunosensor for LC3^+^ EVs was highly sensitive and reliable for practical samples. Furthermore, the detection results were positively correlated with the clinical stages, and the comparison study demonstrated that LC3^+^ EVs were more reliable in distinguishing peripheral blood from cancer patients with different clinical stages than traditional biomarkers, such as CEA and CA15-3. This work opens a new avenue for the exploitation of new biomarkers for breast cancer and provides a new noninvasive detection tool for the early diagnosis and prognosis assessment of breast cancer.

## Experimental section

### Materials and reagents

N-(3-(dimethylaminopropyl)-N^’^-ethylcarbodiimide hydrochloride (EDC), albumin from bovine serum (BSA), N-hydroxysuccinimide (NHS), HAuCl_4_, poly (diallyldimethylammonium chloride) (PDDA), and anti-LC3 antibody produced in rabbit (LC3 cAb) were purchased from Sigma-Aldrich. Anti-LC3 rabbit antibody (LC3 dAb) was purchased from Novus Biologicals. The remaining proteins and antibodies used in this study were purchased from Proteintech Group Inc. Methylene blue (MB) was purchased from Maya Reagent Co., Ltd. Sodium citrate and sodium acetate were purchased from Sangon Biotech (Shanghai, China). Ltd. Graphene oxide (GO) was obtained from Nanjing XFNANO Materials Tech Co. Ltd. FeCl_3_ and ethylene glycol were supplied by Sinopharm Chemical Reagent Co., Ltd. 4-mercaptobutyric acid (C_4_H_8_O_2_S) was purchased from Shanghai Biochempartner Co.,Ltd. 1-butanethiol (C_4_H_10_S) was purchased from Shanghai Aladdin Bio-Chem Tech Co., Ltd. Ultrapure water (18.2 MΩ·cm) was obtained using a Millipore water purification system. The HBcAg ELISA kit was purchased from Shanghai Kehua Bio-Engineering Co. Ltd. Human IL-6, IL-10 ELISA kits, fetal bovine serum (FBS) were purchased from Thermo Fisher Scientific Co., Ltd. RPMI 1640 medium was purchased from HyClone, Cytiva. Penicillin-streptomycin solution and BCA protein assay kits were obtained from Beyotime Biotechnology Co., Ltd. CD9, CD63, TSG101 were purchased from Proteintech Group, Inc. CEA, CA15-3 were purchased from Beijing Key-Bio Biotech Co., Ltd. HER2 was purchased from Novoprotein Scientific Inc. All cell lines were obtained from cell bank of Chinese Academy of Science. BALB/c mice were purchased from Qinglongshan Animal Center. Healthy human, benign breast disease patient and breast cancer patient peripheral blood samples were from Zhongda Hospital.

B16F10, HepG2, EL4, Hepa1-6, MDA-MB-231, and epirubicin-resistant 4T1 cell line (4T1/EPB) cell lines were cultured in RPMI 1640 medium supplemented with 10% FBS and 1% penicillin-streptomycin and grown in a humidified atmosphere at 37 °C with 5% CO_2_.

### Isolation and characterization of LC3^+^ EVs

LC3^+^ EVs from B16F10 cells were prepared as previous report [23]. Briefly, the B16F10 cell culture supernatant was centrifuged at 450 g to remove dead cells and debris. To harvest LC3^+^ EVs released by B16F10 cells, the supernatant was centrifuged at 12,000 g and the sediment was resuspended with PBS and then purified by utilizing LC3-labeled magnetic beads (CST, USA). The total protein concentration of LC3^+^ EVs was determined using a BCA protein assay kit, according to the manufacturer’s instructions. LC3^+^ EVs were then fixed in 1.6% paraformaldehyde, 0.1% ruthenium red, 2.5% glutaraldehyde, 0.064% picric acid, and 100 mM sodium cacodylate, post-fixed with osmium tetroxide and potassium ferricyanide, and embedded in Epon. TEM images were obtained using a JEM 2100 transmission electron microscope (JEOL, Tokyo, Japan). The proteins extracted from LC3^+^ EVs were incubated with the primary anti-LC3 rabbit antibody and exposed to horseradish peroxidase (HRP)-conjugated affinipure goat anti-rabbit IgG. Protein bands were visualized using a Tanon 5200 chemiluminescent imaging system (Tanon, China). LC3^+^ EVs were stained with anti-LC3 rabbit antibody and PE-conjugated goat anti-rabbit IgG (rIgG-PE) and then analyzed using a BD Calibur cytometer (BD, USA).

### Screening for specific anti-LC3 matched antibody pair

Several anti-LC3 antibodies specific to LC3 were selected as candidates. The specificity of the antibody candidates was analyzed using western blot. Proteins extracted from LC3^+^ EVs were separated by SDS-PAGE and transferred to nitrocellulose membranes. The membranes were blocked in 5% skim milk in TBS containing 1% Tween-20 (TBST) at room temperature for 1 h. Afterwards, the membranes were incubated with primary antibodies referred to antibody candidates diluted in 5% skim milk in TBST at 4 °C overnight and exposed to respective HRP-conjugated secondary antibodies at room temperature for 1 h after being washed five times with TBST. Protein bands were visualized using a chemiluminescence assay.

The match between specific anti-LC3 antibodies was verified by flow cytometry. The matching of antibodies indicated antibodies against different antigen epitopes. Briefly, LC3^+^ EVs were stained with rabbit anti-LC3 antibody and subsequently stained with the same or another rabbit anti-LC3 antibody and then incubated with the corresponding rIgG-PE. LC3^+^ EVs stained with rIgG-PE were used as the negative controls.

### Synthesis of GH-MB

For the preparation of GH, 5 mL of a 5 mg mL^− 1^ GO suspension was sonicated for 30 min and then mixed with 2 mL of ethylene glycol. Afterwards, the mixture was sealed in a Teflon autoclave at 180 °C for 12 h, followed by cooling to room temperature. The resulting black hydrogel was rinsed with ultrapure water to obtain the GH.

To prepare GH-MB, GH was sonicated for 30 min and then dispersed in ultrapure water. Subsequently, 4 mL of 1 mg mL^-1^ MB was added to 5 mL of 1 mg mL^-1^ GH, and the mixture was stirred at room temperature for 2 h. Finally, the composite was centrifuged and re-dispersed in ultrapure water.

### Preparation of Fe_3_O_4_-Au composites

The Fe_3_O_4_ nanoparticles were synthesized by a hydrothermal method [33]. Briefly, 0.65 g FeCl_3_ and 0.2 g sodium citrate were dissolvedin ethylenee glycol (20 mL), and 1.2 g sodium acetate was added to themixturey. The mixture was then transferred to a Teflon autoclave at 200 °C for 10 h after vigorous stirring for 30 min at room temperature. The precipitate was collected by centrifugation, washed with ultrapure water and ethanol, and dispersed in ethanol for further use.

Au nanoparticles (AuNPs) were first synthesized for the preparation of the Fe_3_O_4_-Au composites. The AuNPs were synthesized via reduction of HAuCl_4_ according to previous literature [34]. Briefly, 100 mL of 0.01% (w/v) HAuCl_4_ solution was heated to ebullition and 2 mL of 1% sodium citrate was added under stirring. The mixture was then heated for 15 min and cooled for further use.

To prepare Fe_3_O_4_-Au composites, the pH of the Fe_3_O_4_ nanoparticles was adjusted to 9.5, and 1 mL of 1 mg mL^-1^ Fe_3_O_4_ nanoparticles was mixed with 0.4 mL of 4% PDDA solution and then stirred for 30 min at room temperature. The obtained suspension was rinsed with ultrapure water and redispersed in 1 mL of ultrapure water. Finally, 10 mL of AuNPs (pH 7.0) was added to the above solution, followed by stirring at room temperature for 7 h and centrifugation to obtain the precipitate as Fe_3_O_4_-Au composites.

### Preparation of cAb-beads (Fe_3_O_4_-Au-conjugated LC3 cAb)

The Fe_3_O_4_-Au-conjugated LC3 cAb was prepared by covalent binding of the amino groups of the LC3 cAb to the carboxyl groups of the AuNPs. Briefly, 0.1 µL of 4-mercaptobutyric acid was added to 1 mL of 1 mg mL^− 1^ Fe_3_O_4_-Au, and the mixture was stirred overnight at room temperature. After centrifugation, the precipitate was rinsed with ultrapure water and dispersed in ultrapure water. Then 0.1 µL 1-butanethiol was added to the above solution, followed by stirring for 1 h at room temperature. 4-mercaptobutyric acid and 1-butanethiol were used to block the nonspecific binding sites of AuNPs. Then, the mixture was washed with ultrapure water, redispersed into 0.1 M PBS, and mixed with an equal volume of EDC (20 mg mL^− 1^)/NHS (10 mg mL^− 1^) solution to activate the carboxyl groups on the AuNPs. After centrifugation and rinsing, 10 µL of LC3 cAb was added to 1 mL of the resulting solution and the mixture was stirred at 4 °C overnight. Finally, 10 mg of BSA was incubated in the solution at 4 °C for 1 h, and the obtained Fe_3_O_4_-Au-conjugated cAb solution was stored at 4 °C until further use.

### Fabrication of immunosensor and detection of LC3^+^ EVs

Before immunosensor fabrication, the bare glassy carbon electrode (GCE) was polished with 0.3 and 0.05 μm alumina powder and washed ultrasonically in ethanol and ultrapure water. Subsequently, 10 µL GH-MB (1 mg mL^− 1^) was dropped onto the GCE and dried at room temperature for 24 h. Next, 10 µL of EDC (20 mg mL^− 1^)/NHS (10 mg mL^− 1^) solution was added to the electrode for 2 h as a covalent binding agent to link the amino group on LC3 dAb with the carboxyl group on GH. After rinsing with PBS, 10 µL of 10 µg mL^− 1^ LC3 dAb in 0.1 M PBS was dropped onto GCE and incubated at 4 °C overnight. After rinsing, 10 µL of 0.5% (w/v) BSA in 0.1 M PBS was incubated on the GCE at room temperature for 30 min to block nonspecific binding sites. At the same time, different concentrations of LC3^+^ EVs in 0.1 M PBS were incubated with the cAb-beads at 37 °C for 100 min followed by magnetic enrichment, which was denoted as LC3^+^ EVs/cAb-beads. Finally, the LC3^+^ EVs/cAb-beads were dropped onto the GCE and incubated at 37 °C for 60 min. The resulting immunosensor was rinsed with PBS and stored for future use.

Electrochemical measurements were performed in deoxidized 0.1 M PBS solution using a CHI 660E workstation (CHI, USA) with a conventional three-electrode system composed of the modified electrode as the working electrode, platinum wire as the counter electrode, and Ag/AgCl (3 M KCl) as the reference electrode. DPV measurements were carried out from − 0.6 to 0.1 V in deoxidized 0.1 M PBS solution.

### Establishment and treatment of the 4T1/EPB TB mouse model

The 4T1/EPB TB mouse model was established and treated according to our previous study [35]. 4T1/EPB cells in the logarithmic growth phase were digested and resuspended in PBS, the cell concentration was adjusted to 5 × 10^5^ mL^-1^ and the cell suspension was injected subcutaneously into the right breast pad of mice to establish a 4T1/EPB TB mouse model. For the treatment, 4T1/EPB TB mice were vaccinated by subcutaneous injection of normal saline or vaccine containing 30 µg ubiquitinated proteins (UPs) on days 8, 10, and 12 (3 mice per group).

### Enzyme-linked immunosorbent assay (ELISA)

The specificity of the cAb-beads was verified using a human HBcAg ELISA kit, according to the manufacturer’s protocol. HBcAg was used as an interfering protein. Briefly, cAb-beads were incubated with HBcAg, centrifuged, washed, and resuspended in PBS. The above product, PBS, cAb-beads, and HBcAg were cultured in a 96-well plate pre-coated with the capture antibody of HBcAg. After washing off unbound proteins, an HRP-labeled detection antibody of HBcAg was added to the well, which catalyzed the colorimetric reaction with TMB as a substrate and was terminated by H_2_SO_4_. PBS and HBcAg were used as the negative and positive controls, respectively. A microplate reader (Thermo Fisher Scientific, USA) was used to measure the absorbance of the final product at 450 nm.

### Confocal laser scanning microscopy (CLSM)

To confirm the conjugation of LC3 cAb to Fe_3_O_4_-Au, 100 µL of 4-mercaptobutyric acid- and 1-butanethiol-blocked Fe_3_O_4_-Au composites and 100 µL of cAb-beads were mixed with 1 µL of rIgG-PE and incubated for 2 h at room temperature. The precipitates were then removed by magnetic separation, washed three times with PBS, and resuspended in 100 µL of PBS. The above two substances and Fe_3_O_4_-Au composites, cAb-beads were added dropwise on slides and observed by CLSM.

To investigate the binding ability of the cAb-beads to LC3^+^ EVs, 50 µL of LC3^+^ EVs stained with PKH67 (PKH67-labeled LC3^+^ EVs) and 1 µL of LC3 cAb or PBS were incubated at 4 °C for 1 h. The suspensions were then centrifuged at high speed, and the precipitates were obtained after washing three times with PBS. Finally, the precipitates were respectively resuspended in 50 µL of PBS. The above substances were then added to 100 µL of cAb-beads and incubated at room temperature for 2 h. After magnetic separation and washing three times with PBS, the precipitates were resuspended in 100 µL of PBS. The above substances, cAb-beads, and PKH67-labeled LC3^+^ EVs were added dropwise onto the slides and observed using CLSM.

To evaluate the specificity of the cAb-beads, 1 µL of Alexa Fluor 594-conjugated goat anti-mouse IgG (mIgG-594) was added to 100 µL of cAb-beads, 100 µL of cAb-beads and 50 µL of PKH67-labeled LC3^+^ EVs, respectively, and then incubated for 2 h at room temperature. Precipitates were obtained by magnetic separation, washed three times with PBS, and resuspended in 100 µL of PBS. The above substances were added to the slides and observed using CLSM.

### Surface plasmon resonance (SPR) assay

Experiments were performed at 25 ℃ on a BIAcore T200 instrument (GE Healthcare), employing CM5 sensor chips in accordance with the manufacturer’s instruction. Briefly, a total of 50 µL of LC3 protein was combined with a 10 mM sodium acetate solution (pH 5.0), subsequently immobilized onto the CM5 sensor chip surface using the amine-coupling procedure, followed by blocking the remaining activated groups with 1 M ethanolamine (pH 8.5). The adjacent aisle, serving as the reference, underwent a similar activation and blocking procedure, albeit being immobilized with PBS (pH 5.0). To compare the binding affinities of antibodies towards LC3 before and after conjugation through EDC/NHS, antibodies were injected into the flow cell at concentrations of 1, 0.5, 0.25, 0.125, and 0.000625 nM in phosphate buffered saline containing detergent (PBS-P + buffer) (pH 7.4) at a flow rate of 10 uL min^− 1^, allowing for 60 s of association and 90 s of dissociation. Data pertaining to the samples were acquired utilizing the BIAcore T200 Control software (v. 2.0, GE Healthcare). The dissociation constant K_D_ was determined utilizing the BIAcore T200 Evaluation software (GE Healthcare) based on K_D_ = K_d_/K_a_ (where K_a_ is the association rate constant, and K_d_ is the dissociation rate constant), with the K_D_ value being inversely proportional to the antibody affinity [36].

## Results and discussion

### Characterization of LC3^+^ EVs

The morphology of LC3^+^ EVs isolated by differential ultracentrifugation was characterized by TEM, which showed a double-membrane vesicle with sizes ranging from 200 to 900 nm (Fig. S1a). The protein expression and purity of the isolated LC3^+^ EVs were analyzed by western blot and flow cytometry, respectively. As shown in Fig. S1b and S1c, the isolated LC3^+^ EVs expressed a hallmark protein of autophagosomes (LC3) instead of exomes (TSG101and CD63), demonstrating satisfactory purity. It should be noted that LC3^+^ EVs have a single and well-defined surface marker, making them easier to detect in sensing platforms and more suitable as biomarkers.

### Preparation and characterization of cAb-beads

LC3 capture antibody-conjugated Fe_3_O_4_-Au immunomagnetic beads (denoted as cAb-beads) were prepared by covalently linking LC3 capture antibody (LC3 cAb) with Fe_3_O_4_-Au nanocomposites (Fig. 1a). Fe_3_O_4_-Au nanocomposites were successfully synthesized through layer-by-layer assembly of Fe_3_O_4_ nanoparticles (Fig. S2a) and AuNPs with the aid of a PDDA solution under optimized conditions (Fig. S2b), which was confirmed by solution color change and UV-vis absorption analysis (Fig. S2c). The rabbit LC3 antibody with high specificity towards the LC3^+^ EVs, which was used as LC3 cAb, was screened out by western blot (Fig. S3), and the cAb-beads were thus obtained by covalently conjugating the LC3 cAb with Fe_3_O_4_-Au nanocomposites through amido linkage. The affinity of LC3 cAb before and after conjugation with Fe_3_O_4_ was evaluated using surface plasmon resonance (SPR) assay, which showed that the conjugation process had no significant impact on the affinity to the LC3 target (see Fig. S4a,b for more discussion).

**Fig. 2:**
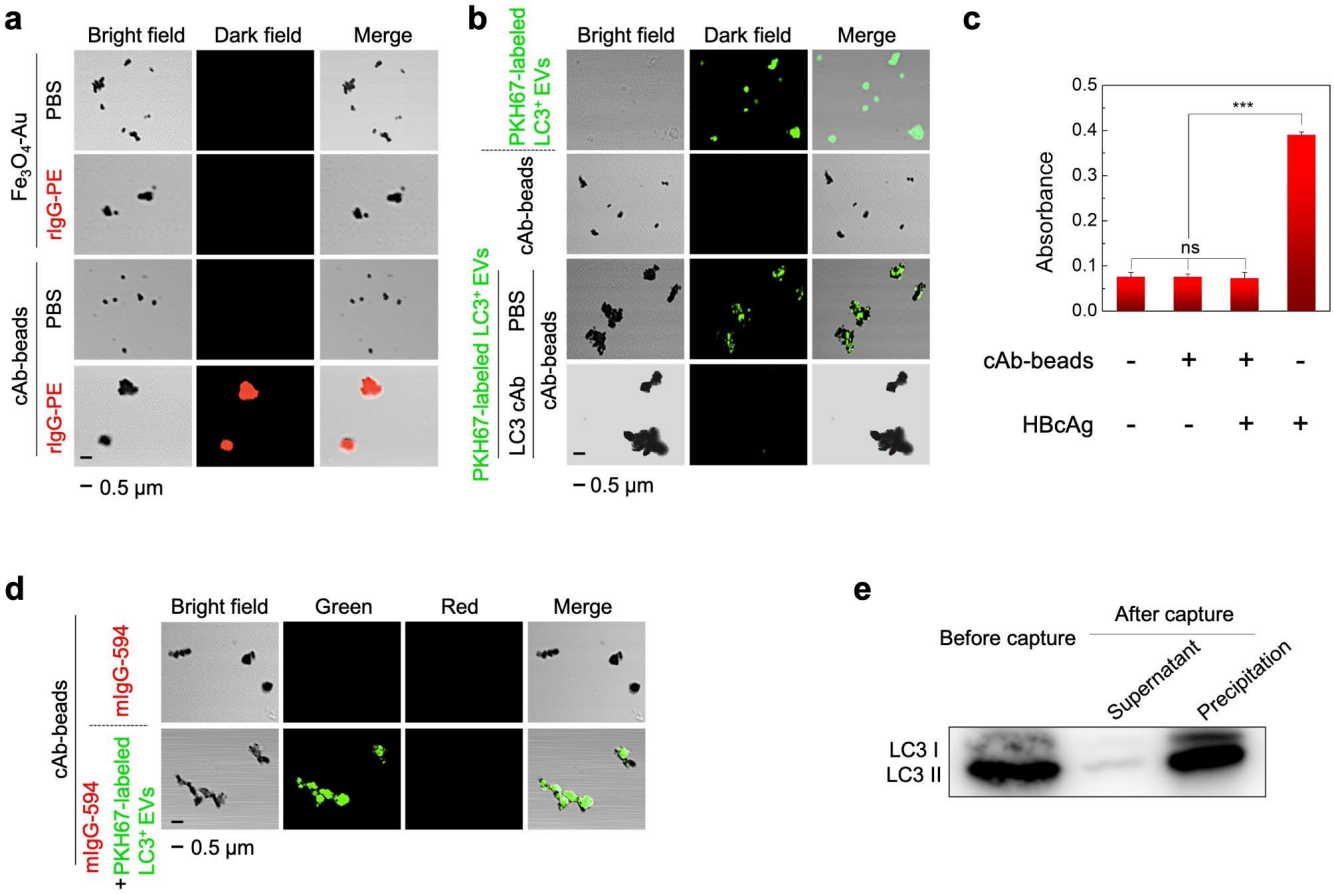
Confirmation of the synthesis of the cAb-beads. (a) CLSM images of Fe_3_O_4_-Au composites and cAb-beads before and after the incubation with red fluorescent rIgG-PE. (b) CLSM images of green fluorescent PKH67-labeled LC3^+^ EVs, cAb-beads, PKH67-labeled LC3^+^ EVs after incubating with cAb-beads, and PKH67-labeled LC3^+^ EVs after incubating with LC3 cAb and cAb-beads successively. (c) The absorbance at 450 nm of TMB from the HBcAg ELISA kit after the test with PBS, cAb-beads, the mixture of cAb-beads and HBcAg, and HBcAg. ^***^p < 0.001, ns: not significant. (d) CLSM images of cAb-beads after the incubation with red fluorescent mIgG-594, and cAb-beads after the incubation with the mixture of mIgG-594 and PKH67-labeled LC3^+^ EVs. (e) Western blot analysis of LC3 proteins (LC3 I and LC3 II) lysized from LC3^+^ EVs, and the supernatant and precipitation after the magnetic separation of LC3^+^ EVs by using cAb-beads. (rIgG-PE: PE-conjugated goat anti-rabbit IgG, mIgG-594: Alexa Fluor 594-conjugated goat anti-mouse IgG)

CLSM was performed to confirm the successful conjugation of LC3 cAb with the Fe_3_O_4_-Au nanocomposites. As shown in Fig. 2a, the cAb-beads could bind to the red fluorescent goat anti-rabbit IgG (rIgG-PE) (Line 4), while Fe_3_O_4_-Au could not (Line 2), revealing that LC3 cAb was successfully conjugated with Fe_3_O_4_-Au nanocomposites. The performance of the cAb-beads, including their ability to capture LC3^+^ EVs, specificity, and enrichment efficiency, was examined. First, after the cAb-beads were co-incubated with green fluorescent PKH67-labeled LC3^+^ EVs, green emission was observed (Line 3 of Fig. 2b). In comparison, the beads without incubation did not show any emission (Line 2 of Fig. 2b), suggesting that the cAb-beads could capture LC3^+^ EVs, probably because of the interaction between LC3^+^ EVs and LC3 cAb on the cAb-beads. To further confirm the binding specificity between LC3^+^ EVs and the cAb-beads, the green fluorescent PKH67-labeled LC3^+^ EVs were first incubated with excess LC3 cAb to block the active binding sites on LC3^+^ EVs, and then incubated with cAb-beads. As shown in Line 4 of Fig. 2b, after magnetic separation, no fluorescence was observed from the cAb-beads, suggesting that PKH67-labeled LC3^+^ EVs after blocking with LC3 cAb would no longer bind to cAb-beads, indicating no nonspecific absorption between the blocked LC3^+^ EVs and the cAb-beads. The specificity of the cAb-beads for LC3^+^ EVs was also explored using an HBcAg ELISA kit (Fig. 2c). Using cAb-beads or cAb-beads after incubation with HBcAg instead of HBcAg for the ELISA test, no evident absorption from the catalytic reaction of the substrate, 3, 3’, 5, 5’-Tetramethylbenzidine (TMB), in the ELISA kit was observed. These results suggest that cAb-beads cannot bind to HBcAg or its antibodies. Meanwhile, as shown in Fig. 2d, after incubating the cAb-beads with a red fluorescent interferent protein, mIgG-594, no red emission was observed. Moreover, after incubation with a mixture of mIgG-594 and green fluorescent PKH67-labeled LC3^+^ EVs, only green emission was observed. These results suggested that the cAb-beads cannot bind to the interfering protein mIgG-594, confirming the excellent specificity of the cAb-beads to LC3^+^ EVs.

To investigate the enrichment efficiency of cAb-beads, they were incubated with a solution containing LC3^+^ EVs. After incubation and subsequent magnetic separation, both the supernatant and the precipitate were lysed. As shown in Fig. 2e, the amount of LC3 protein that was lysed from LC3^+^ EVs in the precipitate was significantly higher than that from the supernatant. Notably, the amount of LC3 protein from the precipitate was quite close to that from the lysate of the original LC3^+^ EVs solution before enrichment, indicating the high capture efficiency of the cAb-beads to LC3^+^ EVs.

### Construction of the immunosensor

**Fig. 3:**
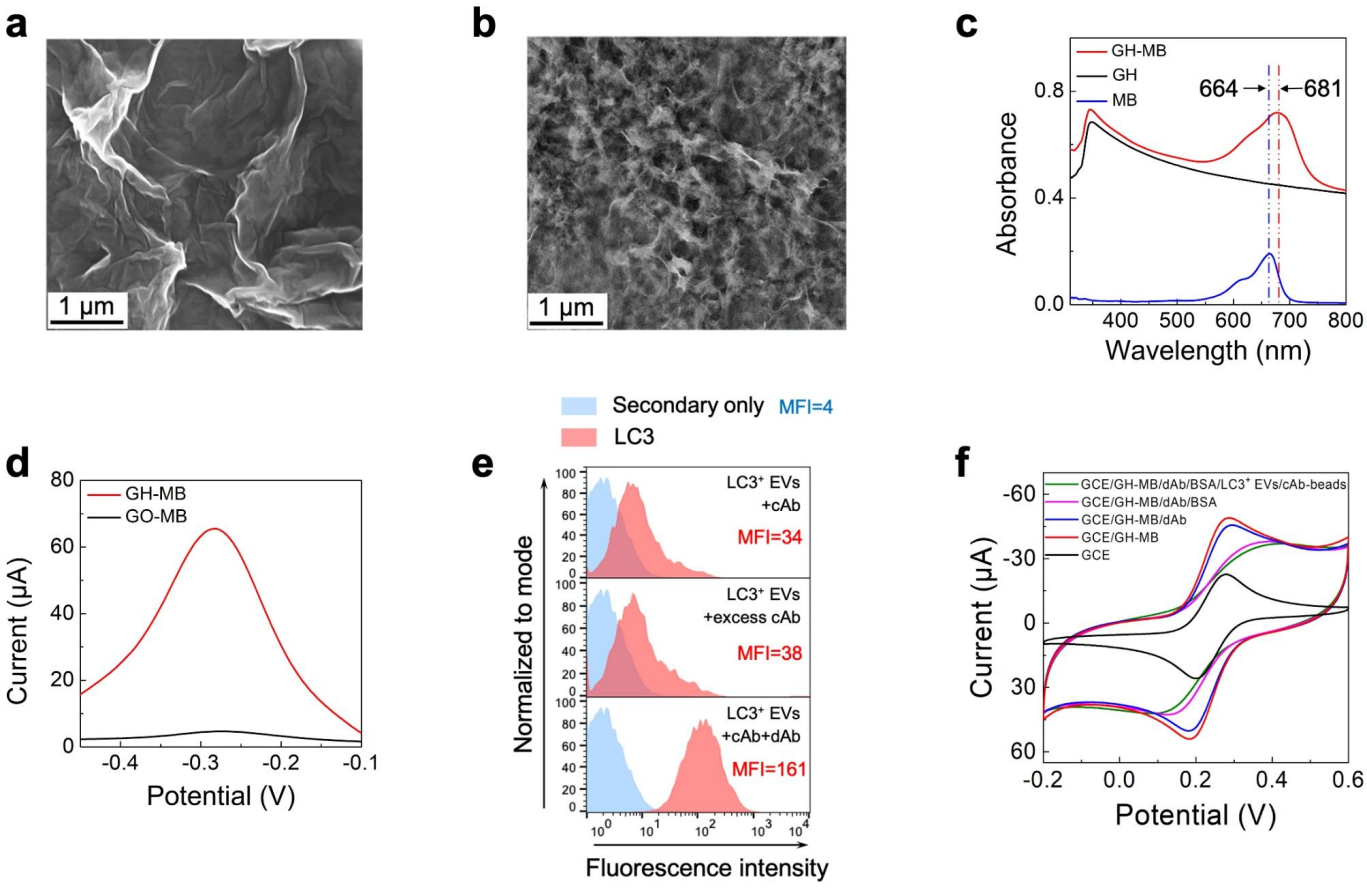
Preparation of GH-MB and establishment of the immunosensor. SEM images of GO (a) and GH (b). (c) UV-vis spectra of GH, MB, and GH-MB. (d) DPV curves of GCEs modified with GO-MB and GH-MB in 0.1 M PBS solution. (e) Flow cytometry analysis of the mixture of LC3^+^ EVs and LC3 cAb, mixture of LC3^+^ EVs and excess LC3 cAb, and mixture of LC3^+^ EVs, LC3 cAb, and LC3 dAb. (f) CV responses of different modified electrodes during immunosensor construction in 0.1 M PBS containing 2 mM K_3_[Fe(CN)_6_]

The immunosensor was constructed using a three-dimensional porous GH-MB composite as the redox probe. As shown in Fig. 3b, the GH displayed well-defined three-dimensional pore with hierarchical structures via π-π interaction and hydrophobic interaction between graphene sheets, which was distinctly different from the planar lamellar structure of GO (Fig. 3a) [37, 38]. The successful synthesis of GH-MB was verified by UV-vis analysis. As shown in Fig. 3c, the GH-MB showed prominent absorption peaks at around 350 and 681 nm, corresponding to the absorption of GH and MB, respectively [39, 40]. Compared to MB alone, the absorption peak of MB in GH-MB was 17 nm redshifted, possibly due to the π-π conjugation between GH and MB [41]. The redox behavior of GH-MB and graphene oxide-methylene blue (GO-MB) was further evaluated by differential pulse voltammetry (DPV), which displayed a pronounced reductive peak centered at -0.3 V (Fig. 3d). In contrast, the peak current of GO-MB was much weaker than that of GH-MB. Notably, the enhanced electrochemical performance of GH-MB could be attributed to the high porosity, enhanced surface area of GH, and enhanced loading of MB.

To construct a sandwich-type immunosensor, another LC3^+^ EVs, LC3 dAb, was screened as a detection antibody using flow cytometry and western blot analysis. As shown in Fig. 3e, the addition of LC3 cAb to LC3^+^ EVs resulted in an increase in the mean fluorescence intensity (MFI) compared to secondary only (from 4 to 34), demonstrating the binding of LC3 cAb to LC3^+^ EVs. Further addition of excess LC3 cAb did not cause an increase in MFI (from 34 to 38), suggesting that all antigenic epitopes for LC3 cAb were occupied. Nevertheless, the subsequent addition of LC3 dAb led to a remarkable promotion of the MFI (from 38 to 161), suggesting that LC3 dAb bound to the specific antigenic epitopes for LC3 dAb on LC3^+^ EVs, unlike LC3 cAb. Western blot analysis did not show any nonspecific bands, indicating the high specificity of both LC3 cAb and LC3 dAb for LC3^+^ EVs (Fig. S3).

The immunosensor was constructed using specific LC3 cAb and LC3 dAb against different antigenic epitopes as capture and detection antibodies, respectively (Fig. 1c). LC3^+^ EVs were first separated and enriched by cAb-beads from the LC3^+^ EV-containing solution (denoted as LC3^+^ EVs/cAb-beads). The construction process was monitored by cyclic voltammetry (CV) in 0.1 M PBS containing 2 mM K_3_[Fe(CN)_6_]. As shown in Fig. 3f, the bare GCE exhibited well-defined redox peaks of [Fe(CN)_6_]^3−^ with an anodic and cathodic peak potential difference of less than 80 mV. After the modification of GH-MB onto GCE, the peak current increased significantly, which may be attributed to the enhanced electron transfer of GH-MB. The peak currents gradually decreased after successive modifications of LC3 dAb, BSA, and LC3^+^ EVs/cAb-beads onto the GCE, revealing that the electron transfer of K_3_[Fe(CN)_6_] on the GCE surface was hindered by these proteins. It was noteworthy that the affinity of LC3 dAb was only slightly reduced after coupling with GH-MB (see more discussion in Fig. S4c,d). These results demonstrated that the immunosensor was successfully constructed.

### Analytical performance of the immunosensor

**Fig. 4:**
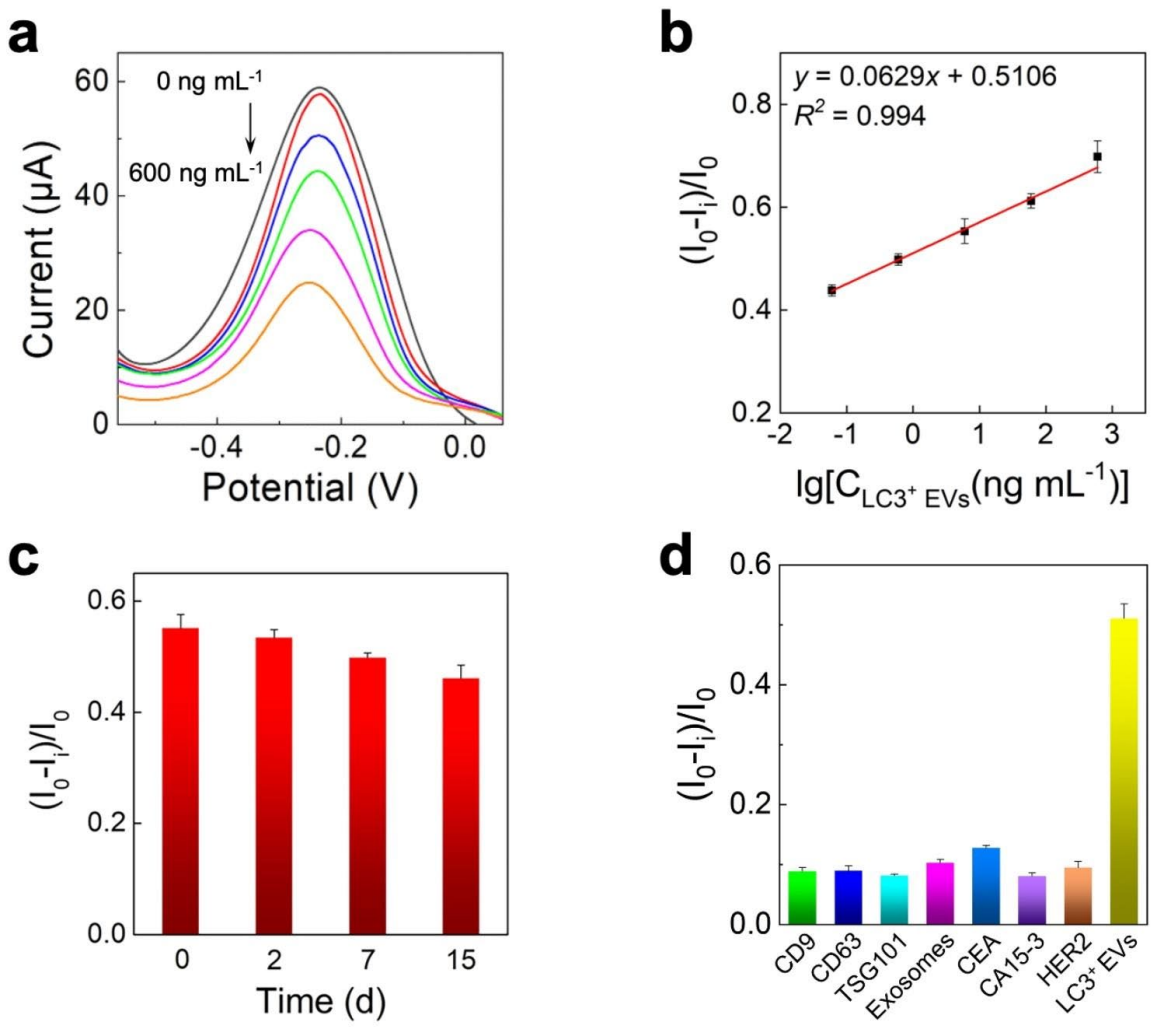
Analytical performance of the immunosensor. The DPV responses (a) and calibration curve (b) of the immunosensor for LC3^+^ EVs with different concentrations in PBS (0.1 M, pH = 7.4). (c) Stability of the immunosensor for 6 ng mL^-1^ LC3^+^ EVs at 4 °C after storage for 0, 2, 7, and 15 days. (d) Responses of the immunosensor to 1 ng mL^-1^ CD9, CD63, TSG101, exosomes, CEA, CA15-3, HER2 and LC3^+^ EVs in PBS (0.1 M, pH = 7.4)

To evaluate the detection performance of the immunosensor, different concentrations of LC3^+^ EVs were assessed using the immunosensor under optimal conditions (Fig. S5). DPV currents decreased gradually when the LC3^+^ EV concentration increased from 0.06 to 600 ng mL^-1^ (Fig. 4a), where an excellent linear relationship (R^2^ = 0.994) was observed between the (I_0_-I_i_)/I_0_ and logarithm values of LC3^+^ EV concentrations in the range of 0.06 to 600 ng mL^-1^ with a limit of detection (LOD) of 15.7 pg mL^-1^ (Fig. 4b). To investigate the stability of the prepared immunosensor, the immunosensors were stored at 4 °C for 2, 7, and 15 days, respectively, and 96.981%, 90.338%, and 83.756% of the initial response for 6 ng mL^-1^ LC3^+^ EVs were retained, indicating that the immunosensor had good stability (Fig. 4c). The selectivity of the immunosensor was investigated by incubating the immunosensor with 1 ng mL^-1^ of CD9, CD63, TSG101, exosomes, CEA, CA15-3, and HER2. As shown in Fig. 4d, the responses to interferents were much lower than those to LC3^+^ EVs, indicating high selectivity of the immunosensor. The reproducibility of the immunosensor was evaluated using five independent electrodes. The variation coefficient was 3.928% (n = 5) for 6 ng mL^-1^ LC3^+^ EVs (Fig. S6), implying that the immunosensor had exceptional reproduction. To further validate the reliability of the immunosensor, the recovery experiment was performed by analyzing three different concentrations (60, 6, and 0.6 ng mL^-1^) of spiked samples in 20 times-diluted healthy human plasma. As shown in Table 1, the recoveries of the spiked samples ranged from 95.659 to 103.808% with a relative standard deviation (RSD) of less than 3.542%, demonstrating high reliability of the immunosensor for quantitative detection of LC3^+^ EVs.

**Table 1:** Recovery of LC3^+^ EVs from the plasma of healthy humans

Samples	Standard value (ng mL^-1^)	Found (ng mL^-1^)	Recovery (%)	RSD (n = 3) (%)
1	60	60.487	100.812	3.542
2	6	5.740	95.659	2.695
3	0.6	0.623	103.808	3.023

### Detection of LC3^+^ EVs in Tumor cell supernatants

To assess the feasibility of the immunosensor for tumor cell samples, the immunosensor was applied to detect LC3^+^ EVs in the supernatant of different tumor cells, including MDA-MB-231 breast cancer, HepG2 liver cancer, EL4 lymphoma, Hepa1-6 liver cancer, and B16F10 melanoma cell lines, which were cultured under hypoxic conditions. Taking B16F10 melanoma cells as examples, a cell number of 10^5^ and a cell culture time of 24 h were selected as the optimized conditions for the culture of tumor cells (Fig. S7a,b). The analysis results for different tumor cell lines by the as-constructed immunosensor showed that all these tumor cells can secrete a significant amount of LC3^+^ EVs (Fig. S7c). These results not only demonstrated that the as-constructed immunosensor can be applied to detect LC3^+^ EVs from tumor cells but also depicted that LC3^+^ EVs could be applied as desirable broad-spectrum tumor markers for different types of cancer diagnosis.

### Application of the immunosensor in mouse models

**Fig. 5:**
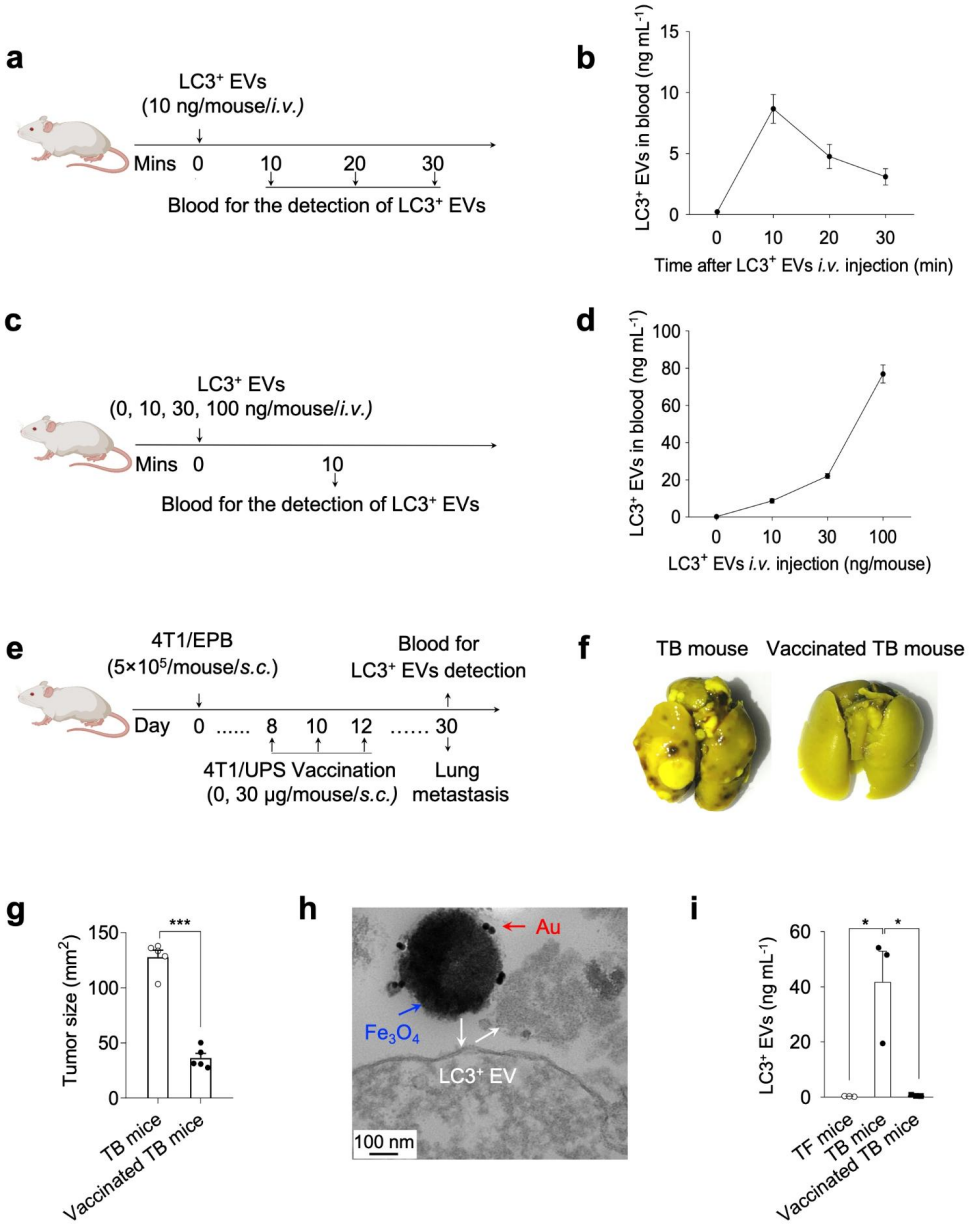
Application of immunosensors in mouse models. Schematic diagram of mouse model construction (a and c). (a) LC3^+^ EVs (10 ng) were intravenously injected into BALB/c mice at 0 min, and peripheral blood was collected at 10, 20, and 30 min for detection. (c) A total of 0, 10, 30, and 100 ng of LC3^+^ EVs was intravenously injected into BALB/c mice, and peripheral blood was collected for detection after 10 min. (b) The concentrations of LC3^+^ EVs from mouse peripheral blood in the mouse model as shown in (a), and (d) the concentrations of LC3^+^ EVs from mouse peripheral blood in the mouse model as shown in (c) measured by the as-constructed immunosensor. (e) Immunization protocol for vaccination. (f) Pulmonary metastatic nodules of the lung tissue in TB mice with or without vaccine treatment. (g) Tumor size in TB mice before and after vaccine treatment. (h) TEM images of LC3^+^ EVs from the peripheral blood of TB mice enriched with cAb-beads. (i) The concentrations of LC3^+^ EVs from the peripheral blood of TF, TB, and TB mice with vaccine treatment detected using the immunosensor. Mouse peripheral blood was centrifuged at a low speed to obtain plasma for testing. ^*^p < 0.05, ^***^p < 0.001. (*i.v.*: intravenous injection, *s.c.*: subcutaneous injection)

To validate the feasibility of the immunosensor for detecting LC3^+^ EVs from mouse peripheral blood, we established mouse models by intravenous injection of LC3^+^ EVs into mice, and LC3^+^ EVs levels in the peripheral blood were determined using the electrochemical immunosensor. Mouse peripheral blood was collected at different times after injection (Fig. 5a), and it was found that the amount of LC3^+^ EVs reached a maximum at 10 min after injection (Fig. 5b). The decrease in LC3^+^ EVs at longer time points could be attributed to the phagocytosis of LC3^+^ EVs by neutrophils [25]. Notably, the concentrations of LC3^+^ EVs detected by the immunosensor increased gradually with the increase in the injected amount of LC3^+^ EVs (Fig.s 5c,d), demonstrating that LC3^+^ EVs from the mouse peripheral blood could be detected with the immunosensor.

To further demonstrate the ability to detect LC3^+^ EVs in TB mice, the immunosensor was applied to compare LC3^+^ EVs levels in TB mice before and after therapy. Our group previously developed a nanovaccine for highly metastatic and highly malignant breast cancer (4T1/EPB) [35]. Accordingly, TB mouse models were built by injecting 4T1/EPB into mice, and the corresponding nanovaccine was then applied for immunization therapy (Fig. 5e). As shown in Fig. 5f, obvious metastatic tumors were observed in the lungs of TB mice, demonstrating successful development of the tumor model. TEM analysis showed that the cAb-beads could separate LC3^+^ EVs from the peripheral blood of TB mice (Fig. 5h), revealing the excellent separation ability of the cAb-beads for LC3^+^ EVs. Furthermore, using the as-constructed immunosensor, a significant amount of LC3^+^ EVs was detected in the peripheral blood of TB mice. In contrast, negligible LC3^+^ EVs were detected in the peripheral blood of the tumor-free (TF) mice (Fig. 5i). After the immunization therapy [35], the amount of LC3^+^ EVs from the peripheral blood of the TB mice decreased significantly (Fig. 5i), which was consistent with the results of immunohistochemistry analysis (Fig. 5f) and tumor growth analysis (Fig. 5g), indicative of powerful anti-tumor effects and high reliability of the immunosensor for the detection of LC3^+^ EVs in TB mice. Thus, it could be concluded that LC3^+^ EVs are reliable tumor markers, and the as-constructed immunosensor was highly feasible for detecting LC3^+^ EVs in TB mice.

### Application of the immunosensor in human peripheral blood

**Fig. 6:**
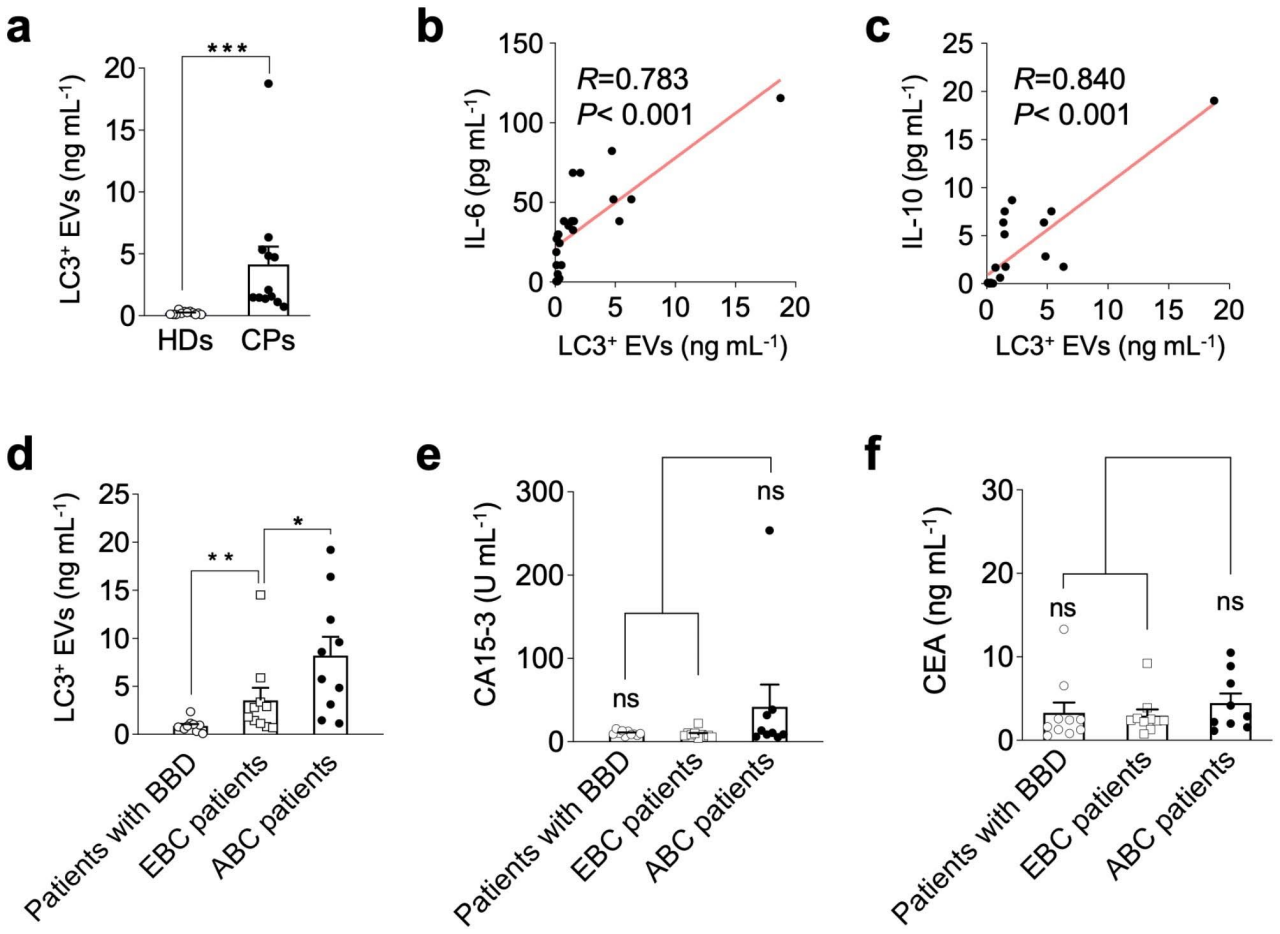
Application of immunosensor to human peripheral blood. (a) The concentrations of LC3^+^ EVs in the peripheral blood of HDs and CPs measured using the immunosensor. Correlation between peripheral blood LC3^+^ EVs levels and IL-6 (b) and IL-10 (c) levels. (d) The concentrations of LC3^+^ EVs in the peripheral blood of patients with BBD, EBC, and ABC measured using the immunosensor. CA15-3 (e) and CEA (f) levels in the peripheral blood of patients with BBD, EBC, and ABC were determined using an ARCHITECT i2000SR immunoassay analyzer. Human peripheral blood was centrifuged at a low speed, and the obtained plasma was used for the above testing. ^*^p < 0.05, ^**^p < 0.01, ^***^p < 0.001, ns: not significant

To verify the practicability of the immunosensor for clinical diagnosis, it was further applied to detect LC3^+^ EVs in the peripheral blood of healthy donors (HDs) and cancer patients (CPs). The clinical characteristics of HDs and CPs are shown in Tables S1 and S2. The electrochemical bioassay results for HDs and CPs displayed distinct differences in LC3^+^ EVs levels (Fig. 6a), indicating the presence of secreted LC3^+^ EVs in the peripheral blood of the CPs. Moreover, LC3^+^ EVs concentrations were positively correlated with the levels of the inflammatory cytokines IL-6 and IL-10, as revealed by ELISA analysis (Fig. 6b,c), demonstrating that LC3^+^ EVs levels were closely related to tumor development and metastasis. Along this line, the electrochemical immunosensor was employed to distinguish patients with benign breast disease (BBD) and breast cancer of different stages including early breast cancer (EBC) and advanced breast cancer (ABC). As shown in Fig. 6d, immunosensing results detected by the electrochemical immunosensor displayed distinct variability in the abundance of LC3^+^ EVs in patients with BBD, EBC, and ABC. In sharp contrast, there was no obvious difference in the levels of traditional breast cancer biomarkers, for example, CA15-3 and CEA, for patients with BBD, EBC, and ABC, as determined by the ARCHITECT i2000SR immunoassay analyzer (Fig. 6e,f). These results further demonstrated that LC3^+^ EVs levels in peripheral blood may be suggestive of early and staged diagnosis of breast cancer. Therefore, all these results indicated that LC3^+^ EVs might be more desirable and reliable cancer biomarkers than traditional cancer biomarkers, and the as-constructed immunosensor was applicable for clinical applications.

## Conclusion

In this study, for the first time, LC3^+^ EVs were identified as biomarkers for breast cancer diagonosis with an electrochemical immunosensor using three-dimensional GH-MB as a redox probe. By using a simple magnetic separation technique, LC3^+^ EVs could be easily captured and separated from the tumor cell supernatant and peripheral blood. Benefiting from the high porosity, large surface area, and abundant binding sites of GH, the as-fabricated immunosensor displayed reliable detection of LC3^+^ EVs over a wide range with a low detection limit of 15.7 pg mL^− 1^. The LC3^+^ EVs levels in different samples including tumor cell supernatants, peripheral blood of TB mouse models before and after immune therapy, and breast cancer patients with different subtypes and stages were determined with the electrochemical immunosensor. Distinct differences in LC3^+^ EVs levels were observed in TB mouse models before and after immunotherapy, and the expression levels of LC3^+^ EVs for benign breast disease and breast cancer were clearly distinguished. More importantly, the subtypes of breast cancer, that is, early and advanced breast cancer could be successfully discriminated according to the expression levels of LC3^+^ EVs. Our work demonstrated that LC3^+^ EVs could be applied as new reliable biomarkers for breast cancer, which provided a new avenue for the non-invasive early diagnosis.

### Electronic supplementary material

Below is the link to the electronic supplementary material.

Supplementary Material 1

## Data Availability

All data generated or analyzed during the study are included in the article.
