# Supplementary material for: Identifying tumor cell-released extracellular vesicles as biomarkers for breast cancer diagnosis by a three-dimensional hydrogel-based electrochemical immunosensor
Source: J Nanobiotechnology. 2023 Dec 7;21:467. doi: 10.1186/s12951-023-02180-y (PMC10701998; doi:10.1186/s12951-023-02180-y)
Supplement: Supplementary file 1 — Supplementary Material 1 [file 12951_2023_2180_MOESM1_ESM.docx]

**Supplemental Material**

**Identifying Tumor Cell-released Extracellular Vesicles as Biomarkers for Breast Cancer Diagnosis by a Three-dimensional Hydrogel-based Electrochemical Immunosensor**

Yue Zhang^1,2^, Deng Pan^2^, Zhenqiang Ning^2^, Fang Huang^2^, Yiting Wei^2^, Mingming Zhang^2^, Yuanjian Zhang^3^, Li-xin Wang^2*^, Yanfei Shen^2*^

^1^Clinical Medical Laboratory Center, The Affiliated Taizhou People’s Hospital of Nanjing Medical University, Taizhou 225300, China

^2^Medical School, Southeast University, Nanjing 210009, China

^3^School of Chemistry and Chemical Engineering, Southeast University, Nanjing 211189, China

^*^Address correspondence to: Li-xin Wang; [lxwang@seu.edu.cn](mailto:lxwang@seu.edu.cn) and Yanfei Shen; [Yanfei.Shen@seu.edu.cn](mailto:Yanfei.Shen@seu.edu.cn).


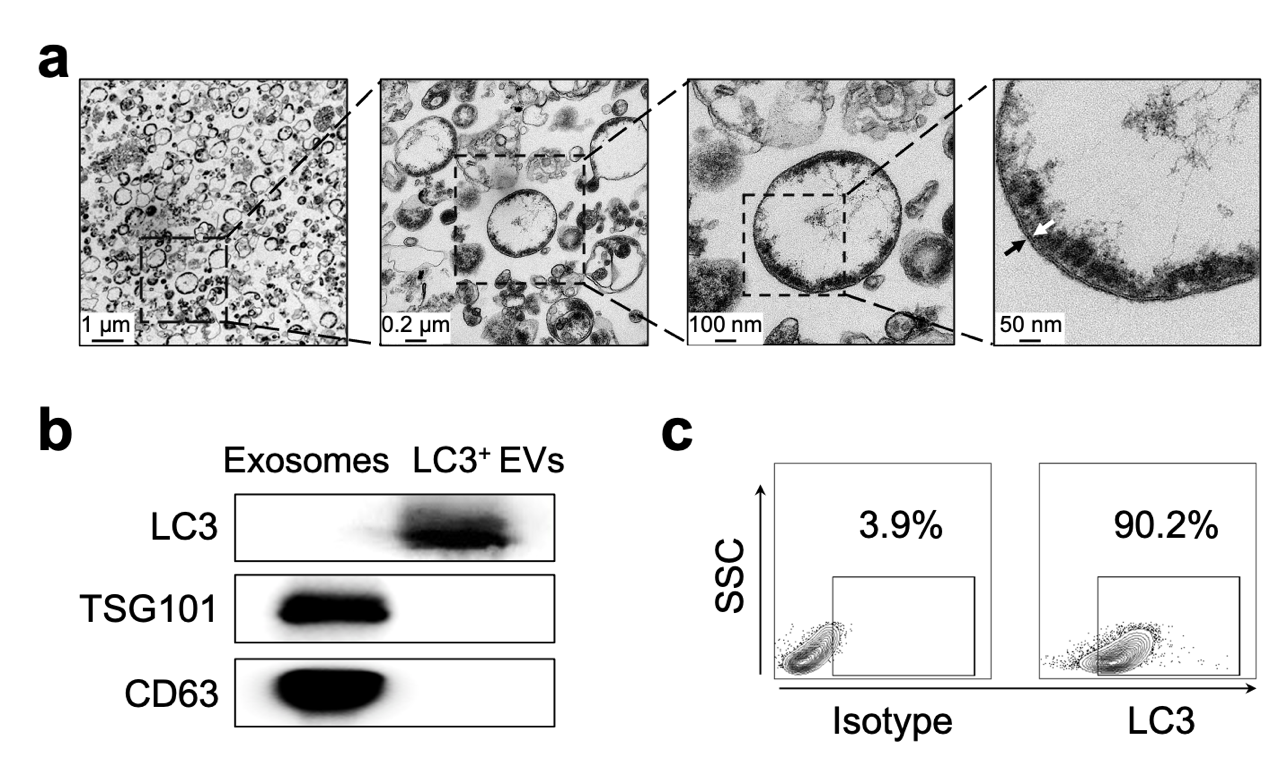


**Fig. S1** Characterization of LC3^+^ EVs. (a) TEM images of collected LC3^+^ EVs. (b) Western blot analysis of exosomes and LC3^+^ EVs. (c) Flow cytometry analysis of LC3 proteins in the collected LC3^+^ EVs.


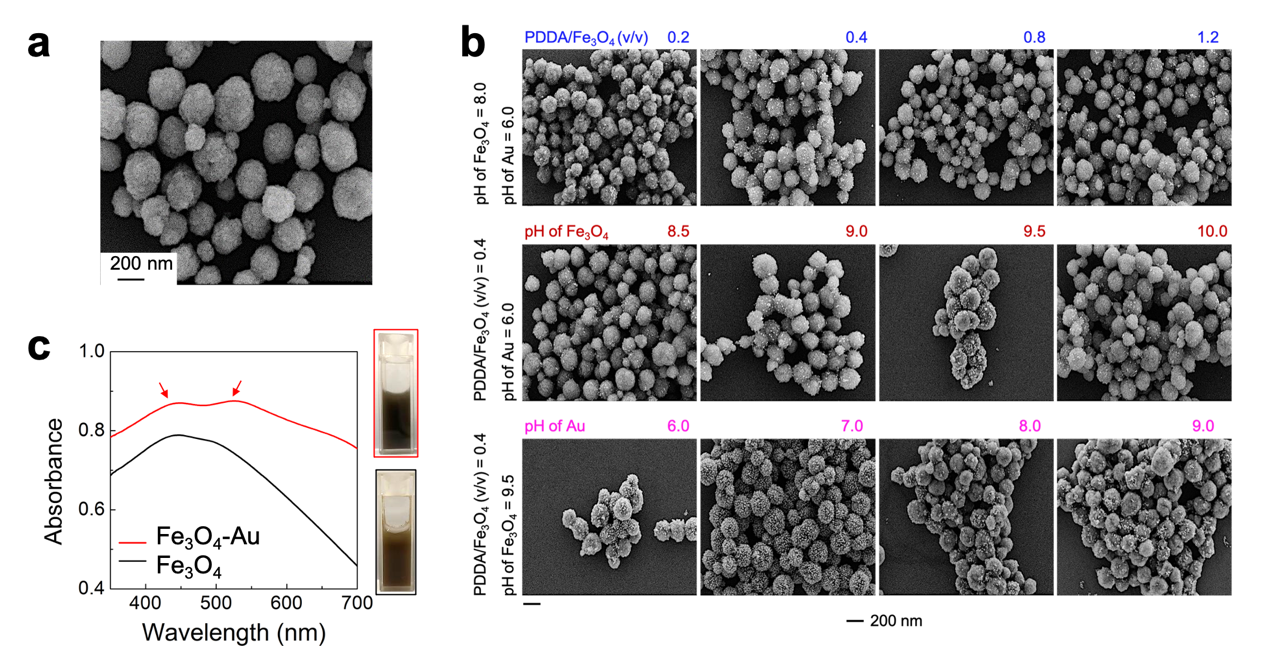


**Fig. S2** Establishment of Fe_3_O_4_-Au composites. (a) SEM image of Fe_3_O_4_ nanoparticles. (b) SEM images of Fe_3_O_4_-Au composites prepared in different conditions. Line 1: SEM images of Fe_3_O_4_-Au composites prepared with different volume ratios of PDDA to Fe_3_O_4_ solution when Fe_3_O_4_ solution at pH = 8.0 and Au solution at pH = 6.0. Line 2: SEM images of Fe_3_O_4_-Au composites prepared with Fe_3_O_4_ solutions with different pH values when volume ratios of PDDA to Fe_3_O_4_ solution was 0.4 and Au solution at pH = 6.0. Line 3: SEM images of Fe_3_O_4_-Au composites prepared with Au solutions with different pH values when volume ratios of PDDA to Fe_3_O_4_ solution was 0.4 and Fe_3_O_4_ solution at pH = 9.5. (c) UV-vis spectra of Fe_3_O_4_ nanoparticles and Fe_3_O_4_-Au composites. The black and red frames are the photos of Fe_3_O_4_ nanoparticles and Fe_3_O_4_-Au composites, respectively.


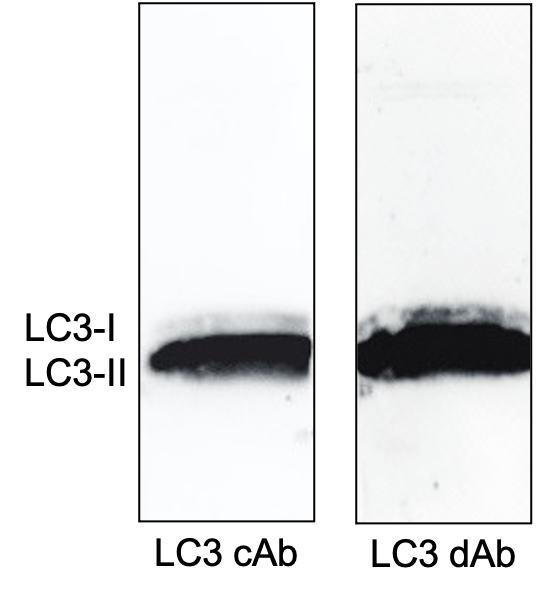


**Fig. S3** Western blot analysis of LC3 cAb and LC3 dAb.

**
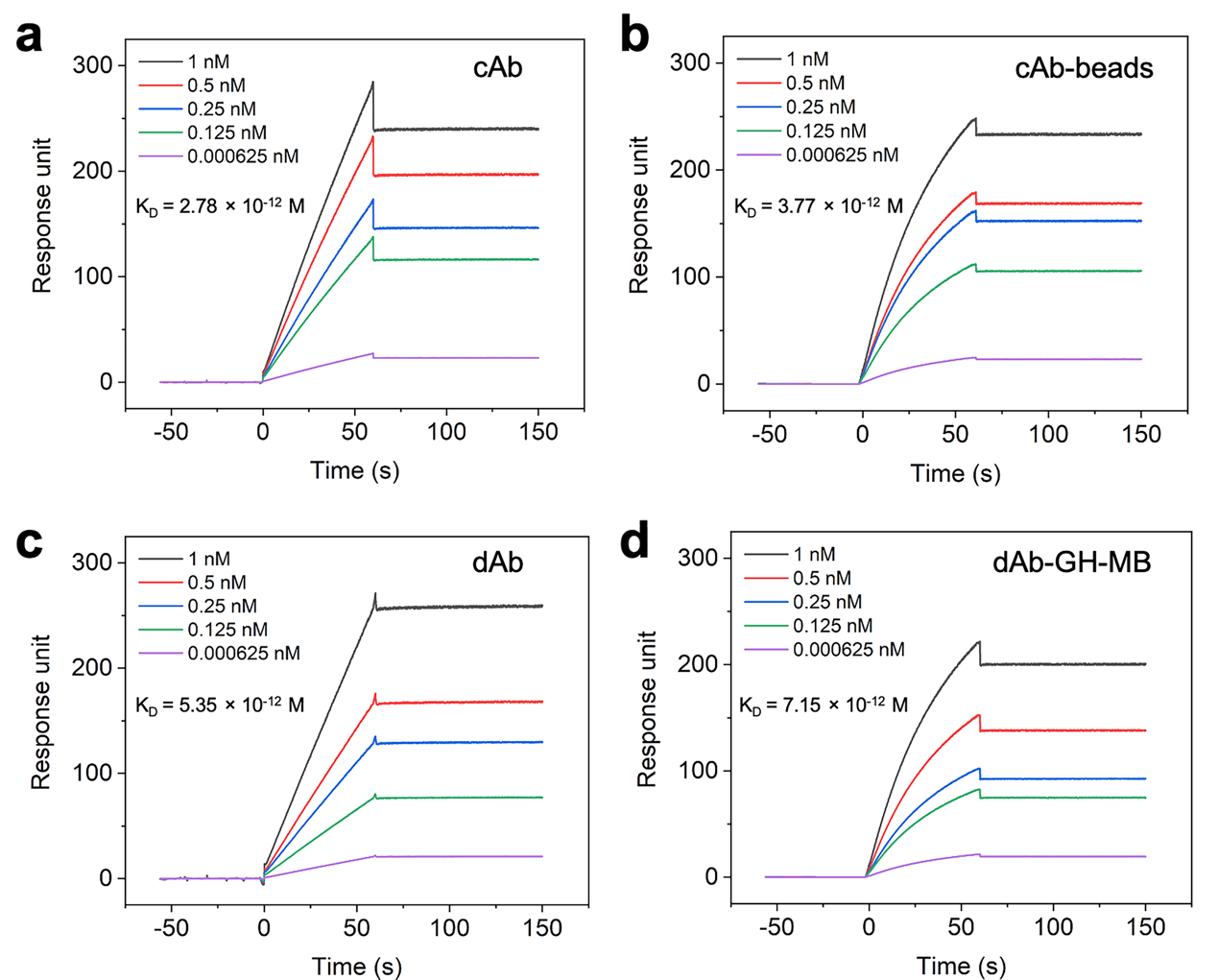
Fig. S4** SPR of (a and b) cAb and (c and d) dAb binding to LC3 protein before and after conjugation through EDC/NHS. dAb-GH-MB was the product of dAb coupled to GH-MB by EDC/NHS followed by blocking using BSA. LC3 proteins were immobilized onto CM5 sensor chips and tested for binding with gradient concentrations of antibodies.

As shown in Fig. S4a and S4b, the dissociation constants (K_D_) of cAb before and after conjugation via EDC/NHS were 2.78 × 10^-12^ M and 3.77 × 10^-12^ M, respectively, suggesting a slight reduction in affinity after the conjugation. However, the affinity still remained high [1, 2]. Similarly, the K_D_ values of dAb before and after conjugation to GH-MB via EDC/NHS were 5.35 × 10^-12^ M and 7.15 × 10^-12^ M, respectively, indicating that EDC/NHS had a minimal effect on the affinity of dAb (Fig. S4c and S4d). In summary, the conjugation process had a negligible impact on the affinity of the antibodies, with only a slight reduction in affinity observed. Thus, the antibodies retained their high affinity (K_D_ values in the picomolar range), which was conducive to the specific capture of cAb-beads to LC3^+^ EVs and the immunosensor construction [3].


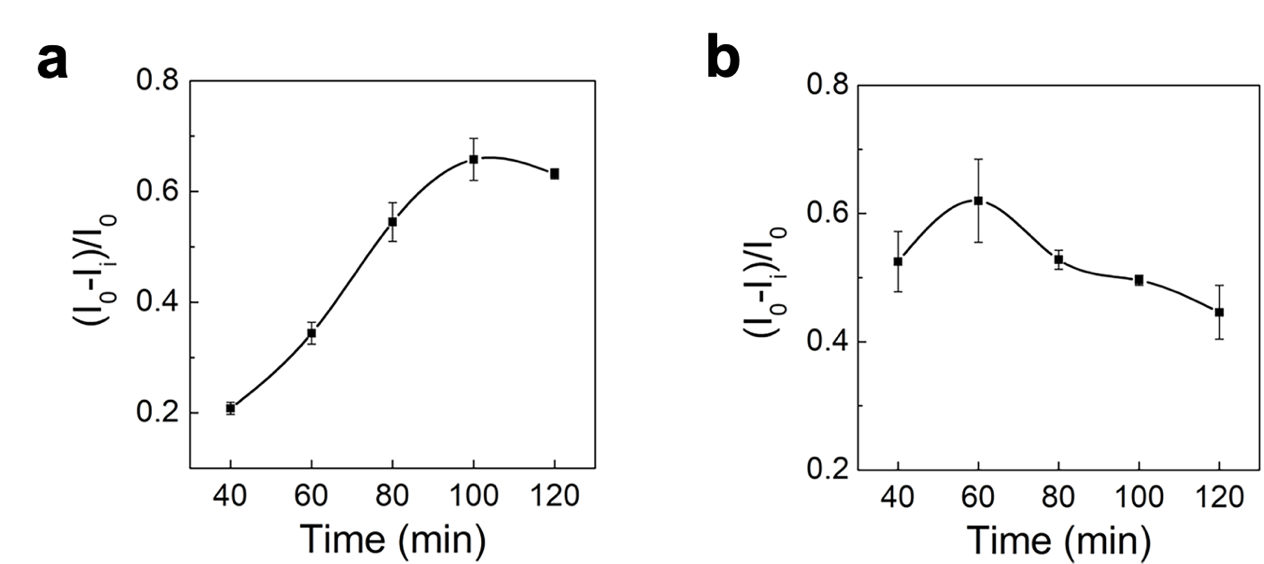


**Fig. S5** Optimization of the immunosensor.

The incubation times for cAb-beads with LC3^+^ EVs (a) and LC3^+^ EVs/cAb-beads with LC3 dAb (b) were optimized at 100 and 60 min, respectively.


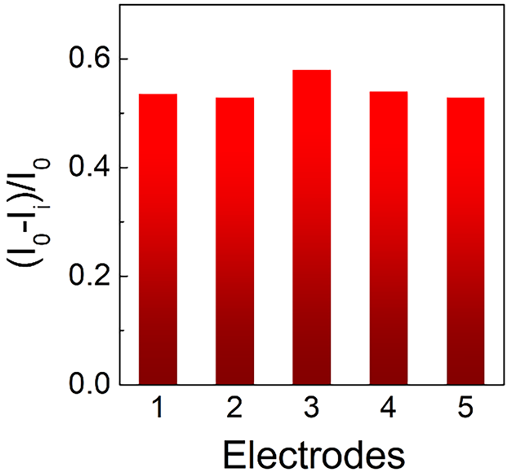


**Fig. S6** Ratio values of immunosensor for 6 ng mL^-1^ LC3^+^ EVs with different electrodes.


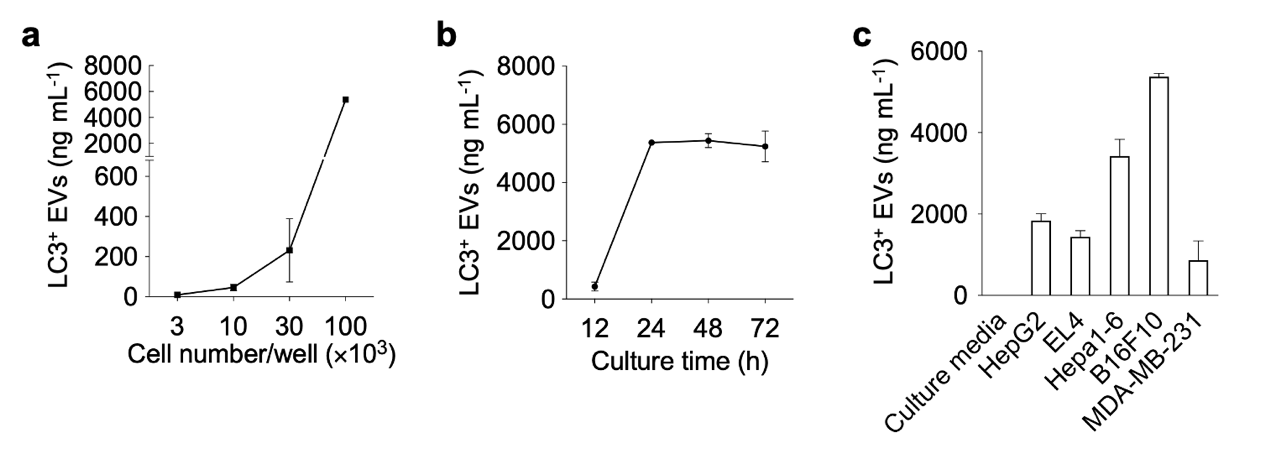


**Fig. S7** Detection of LC3^+^ EVs from tumor cell supernatants using the immunosensor. Detection of LC3^+^ EVs in the supernatant of B16F10 melanoma cells cultured with different cell numbers (a) and culture times (b). (c) Detection of LC3^+^ EVs in the supernatants of different tumor cell lines.

**Table S1.** Clinical characteristics of 12 cancer patients (Fig. 6a).

| **No.** | **Age/Sex** | **Primary tumor site** | **Histologic diagnosis** | **Stage** |
| --- | --- | --- | --- | --- |
| 01 | 40/M | Colon | Adenocarcinoma | II |
| 02 | 62/F | Colon | Adenocarcinoma | III |
| 03 | 61/F | Lung | Adenocarcinoma | IV |
| 04 | 71/F | Lung | Adenocarcinoma | IV |
| 05 | 46/M | Lung | Adenocarcinoma | IV |
| 06 | 69/M | Lung | Adenocarcinoma | IV |
| 07 | 65/M | Stomach | Adenocarcinoma | III |
| 08 | 59/M | Stomach | Adenocarcinoma | III |
| 09 | 60/M | Larynx | Squamous carcinoma | II |
| 10 | 52/M | Esophagus | Squamous carcinoma | I |
| 11 | 77/M | Esophagus | Squamous carcinoma | III |
| 12 | 41/F | Breast | Adenocarcinoma | II |

**Table S2.** Clinical characteristics of 10 patients with benign breast disease (Fig. 6d-f).

| **No.** | **Age/Sex** | **Clinical diagnosis** |
| --- | --- | --- |
| 01 | 45/F | Fibroadenoma |
| 02 | 45/F | Fibroadenoma |
| 03 | 58/F | Accessory breast |
| 04 | 37/F | Mastosis with intraductal papilloma |
| 05 | 67/F | Mastosis with fibroadenoma |
| 06 | 26/F | Fibroadenoma |
| 07 | 48/F | Fibroadenoma |
| 08 | 52/F | Fibroadenoma |
| 09 | 57/F | Mastosis with fibroadenoma |
| 10 | 51/F | Fibroadenoma |

**Table S3.** Clinical characteristics of 10 patients with early breast cancer (Fig. 6d-f).

| **No.** | **Age/Sex** | **Histologic diagnosis** | **Stage** |
| --- | --- | --- | --- |
| 01 | 52/F | Invasive ductal carcinoma | II |
| 02 | 48/F | Ductal carcinoma in situ | 0 |
| 03 | 45/F | Invasive ductal carcinoma | II |
| 04 | 68/F | Invasive ductal carcinoma | II |
| 05 | 54/F | Invasive ductal carcinoma | II |
| 06 | 56/F | Invasive ductal carcinoma | II |
| 07 | 64/F | Invasive ductal carcinoma | I |
| 08 | 51/F | Invasive ductal carcinoma | II |
| 09 | 51/F | Invasive ductal carcinoma | II |
| 10 | 49/F | Invasive ductal carcinoma | II |

**Table S4.** Clinical characteristics of 9 patients with advanced breast cancer (Fig. 6d-f).

| **No.** | **Age/Sex** | **Histologic diagnosis** | **Stage** |
| --- | --- | --- | --- |
| 01 | 65/F | Invasive ductal carcinoma | III |
| 02 | 55/F | Invasive ductal carcinoma | III |
| 03 | 59/F | Invasive ductal carcinoma | II-III |
| 04 | 55/F | Invasive ductal carcinoma | III |
| 05 | 66/F | Invasive ductal carcinoma | III |
| 06 | 78/F | Invasive ductal carcinoma | IV |
| 07 | 62/F | Invasive ductal carcinoma | IV |
| 08 | 35/F | Invasive ductal carcinoma | III |
| 09 | 58/F | Invasive ductal carcinoma | IV |

**References**

1. Yu X, Orr CM, Chan HTC, James S, Penfold CA, Kim J, Inzhelevskaya T, Mockridge CI, Cox KL, Essex JW, et al. Reducing affinity as a strategy to boost immunomodulatory antibody agonism. Nature. 2023;614:539-547.

2. Guo X, Zhang D, Wang Z, Xu S, Batistic O, Steinhorst L, Li H, Weng Y, Ren D, Kudla J, et al. Cold-induced calreticulin OsCRT3 conformational changes promote OsCIPK7 binding and temperature sensing in rice. EMBO J. 2023;42:e110518.

3. Borgia A, Borgia MB, Bugge K, Kissling VM, Heidarsson PO, Fernandes CB, Sottini A, Soranno A, Buholzer KJ, Nettels D, et al. Extreme disorder in an ultrahigh-affinity protein complex. Nature. 2018;555:61-66.
